# Supplementary material for: Perceived risk of type 2 diabetes: Using linked genomic, clinical and questionnaire data to understand the potential use of genetic risk tools in British South Asians
Source: PLOS Glob Public Health. 2025 Mar 31;5(3):e0004274. doi: 10.1371/journal.pgph.0004274 (PMC11957276; doi:10.1371/journal.pgph.0004274)
Supplement: S2 Appendix — (DOCX) [file pgph.0004274.s002.docx]

S2 Appendix. Recruitment process in Genes & Health and the present study.

Genes & Health takes a 2-stage recruitment process. In Stage 1, British Bangladeshi and British Pakistani individuals aged 16 and above—who are living in, working in or within reach of local communities in East London, Bradford and Manchester—are invited to participate. A convenience sampling approach is taken, with bilingual researchers recruiting volunteers from settings such as local mosques, libraries, GP surgeries and outpatient clinics. Stage 1 volunteers complete a brief baseline questionnaire, donate saliva samples for DNA extraction and genetic tests, provide consent for researchers to access their EHRs, as well as consent to be recontacted (up to four times per year) for Stage 2 recall studies.

Under these approvals, Stage 2 procedures in Genes & Health offer the opportunity to invite volunteers for more detailed study visits—e.g. for clinical assessment and/or the collection of biological samples, recall-by-genotype and/or phenotype—and also to develop trials-within-cohorts or sub-cohorts. These studies are, however, subject to separate ethics approvals—as well as volunteer acceptability and Genes & Health Community Advisory Group approvals. The present study takes place under such Stage 2 procedures.

Guided by the inclusion criteria set out for the questionnaire, we identified eligible volunteers using their linked and pseudonymised demographic and health data stored in Genes & Health, as of the July 2022 data release (S1 Fig).


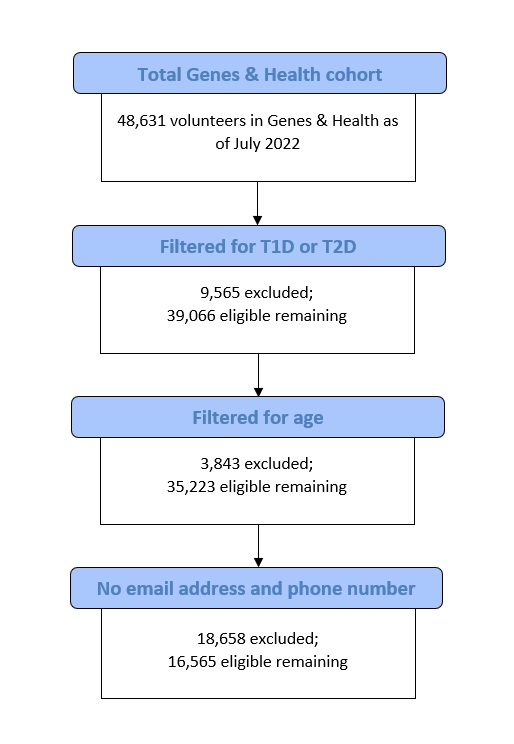


S1 Fig. Flowchart illustrating eligibility screening process.

Based on the original paper from which the primary intention outcome in our questionnaire was extracted, the authors estimated that a minimum sample size of 385 was needed to reach a power of 80%—with a 5% margin of error, a confidence level of 95% and SD of 0.05 [1]. To ensure balanced representation across four different age groups in the present study—i.e. 16 to 25 years; 26 to 35 years; 36 to 45 years and 46 to 59 years—it was agreed that at least 97 (385/4) participants in every age group was needed. Given the exploratory nature of the study, the final target of recruitment was set at approximately 120 participants per age group.

This was the first large-scale online questionnaire study conducted in Genes & Health—hence there were no available estimates surrounding possible response rates. In the first instance, we undertook a stratified random sampling approach to study recruitment. One thousand eligible volunteers were randomly selected according to each age group and sex strata—and then securely extracted from the Genes & Health TRE. In this initial phase, about 20% of all invitations could not be delivered to participants due to inactive email addresses and/or phone numbers. Of the invitations successfully sent, a total of 133 complete responses were collected—providing a response rate of approximately 17% (133/800). There were 51 (38%), 31 (23%), 33 (25%) and 18 (14%) responses from the youngest to oldest age groups, respectively.

Based on these figures, it was determined that an additional 4,000 eligible participants would be extracted from the TRE for the next phase of recruitment. Additionally, stratification was weighted for more participants in the older age groups—since the first phase of recruitment indicated progressively lower response rates from the youngest to oldest age groups. The breakdown of proportions in the participants extracted from the TRE for this second phase of recruitment was as follows—500 (12.5%), 750 (18.7%), 750 (18.7%) and 2000 (50%)—from the youngest to oldest age groups, respectively. Participants were again randomly selected within each age strata—and according to equal sex representation.
