# Supplementary material for: Perceived risk of type 2 diabetes: Using linked genomic, clinical and questionnaire data to understand the potential use of genetic risk tools in British South Asians
Source: PLOS Glob Public Health. 2025 Mar 31;5(3):e0004274. doi: 10.1371/journal.pgph.0004274 (PMC11957276; doi:10.1371/journal.pgph.0004274)
Supplement: S3 Appendix — (DOCX) [file pgph.0004274.s003.docx]

S3 Appendix. Questionnaire items.

The following provides an overview of the items included in the study questionnaire. The complete questionnaire as presented on REDCap—including the participant information sheet and consent form—can be found further below.

*Knowledge of the genetic basis of T2D*

This was assessed using a newly established set of measures—rigorously developed in Genes & Health volunteers alongside a clinical geneticist, as part of a simultaneous study on genetic literacy conducted in Genes & Health. Feedback from the Genes & Health research team indicated this to be a measure relevant and important for the purposes of the present study. It contains five items, each measured on a five-point scale—and higher scores indicate higher levels of knowledge of the genetic basis of T2D.

*Perceived risk for T2D*

This was evaluated using a single-item measure, inviting participants to rate their thoughts on their personal risk of getting T2D in their lifetime on a four-point scale (almost no risk; a slight risk; a moderate risk; a high risk). It was adapted from a measure of perceived risk that has already been widely used in the literature examining genetic risk perceptions for common conditions, including T2D [1]. Higher scores on this measure reflect higher perceived risk.

*Interest in genetic testing*

This section began with a short excerpt explaining the nature and purpose of genetic tests in the context of T2D (details below). Participants were then asked to answer a range of questions about their interest in getting these tests themselves. Questions included the likelihood and importance for them to receive genetic tests for T2D—which were adapted from [2, 3]. Higher scores on these items indicate greater levels of interest in genetic testing for T2D. We included a follow up question asking participants who were interested in receiving genetic tests to report the age at which they would prefer to find out, if they had high genetic risk for T2D. Additionally, there was a separate item asking participants to report if they have ever personally paid for a private genetic test to find out about their heritage or ancestry. This was taken to further reflect participants’ general levels of interest in getting genetic tests.

*Perceived benefits of genetic testing*

This was measured using a set of six items adapted from [3]. The statements included both positive and negative outcomes of genetic testing—e.g. “Finding out about my genetic risk for developing type 2 diabetes would help me make important decisions about my health”; “Getting a genetic test would be a frightening or stressful experience for me”. Negative statements were reverse scored. All items were measured on a four-point scale (strongly disagree; disagree; agree; strongly agree)—and higher scores reflect greater perceived benefits of genetic testing.

*Perceived control*

Measurements of perceived control were adapted from a range of questions extracted from [1, 2]. There were four items, again covering both positive and negative statements—e.g. “Overall, I feel that I am able to control my health”; “If a genetic test tells me that I have an above-average risk for type 2 diabetes, then I would think that type 2 diabetes cannot be prevented”. Negative statements were reverse scored. Items were also measured on a four-point scale (strongly disagree; disagree; agree; strongly agree)—where higher scores reflect greater perceived control.

*Familial variables*

The next section of the questionnaire included a range of familial variables—including questions on participants’ known family members and/or close social contacts with T2D history. Here, traditional measures assessing family history information were expanded upon to include participants’ extended family members and/or close social contacts as potential options. These included aunts, uncles, cousins, as well as “unofficial” family members such as family friends—in acknowledgement of larger familial and social networks within South Asian communities that can play important roles in informing individuals’ conceptions of risk and disease [4-6]. This wider range of options was strongly supported by volunteers with whom our questionnaire was workshopped.

The next questions were in relation to household information and family health behaviours—the latter of which was adapted from a version of the UK Diabetes and Diet Questionnaire which has been optimised and validated in South Asians [7, 8]. Through our PPI sessions, a final measure containing eight items was finalised, encompassing a range of lifestyle factors such as diet and physical activity. Higher scores on these items reflect healthier family health behaviours (some statements were reverse scored).

*Outcome variables*

The primary outcome measure of intention was adapted from existing work on T2D risk perceptions in minoritised ethnic groups in the United States [9]. Three items measured on four-point scales were included to capture participants’ intention to adopt health behaviours that can prevent or delay T2D, if a genetic test shows that they are at above-average risk for T2D. These encompass participants’ likelihood of seeking advice from healthcare professionals, increasing physical activity, as well as improving dietary habits. Higher scores indicate stronger self-reported intention.

Additionally, a secondary measure of intention was included in the questionnaire—asking participants if they would like to receive an email about further online resources on health behaviours that can prevent or delay T2D. A positive response on this item was taken as a further indicator of participants’ willingness and readiness to engage in preventative health behaviours.

| **DNA & Future Risk of Diabetes**  Thank you for your interest in this study!  Please read the information below carefully.  British Bangladeshis and British Pakistanis have some of the highest rates of type 2 diabetes in the UK—and you may be personally affected by the condition. Genes—which contain DNA that individuals inherit from their parents—can be linked to a person’s risk of health conditions such as type 2 diabetes. In the future, the NHS might be able to give people DNA/genetic information about their health, so they get an earlier warning of their type 2 diabetes risk. However, we don't know if people would want to have this information given to them—or if they know what this risk means for them.  This survey will be a first step to help us understand how British Bangladeshis and British Pakistanis think about their risk of type 2 diabetes in the DNA. For example, people may have different beliefs about the level of personal control they have over their health, after receiving DNA information. We want to study how these beliefs are affected by age and experience of type 2 diabetes (e.g. in family members). For example, if a person already has a close a family member affected by type 2 diabetes, they may react differently to DNA information about their own health. If you would like to take part in this survey, please tick the check box below. If you have any specific questions or concerns, please contact the Genes & Health research team at [elgh@qmul.ac.uk](mailto:elgh@qmul.ac.uk).  Thank you very much for your time.  I confirm that I have read and understood the information above. I would like to take part in the study. |
| --- |

**Part 1 of 10**

**Welcome and thank you for agreeing to take part in this study about DNA & Future Risk of Diabetes!**

**Your responses will be stored securely and anonymously. Any information you give us will remain confidential and it will only be used for the purposes of this survey.**

**Please answer the questions below to check if you meet the criteria for this study:**

| Please select your age group (in years): | Below 16  16 – 29  30 – 39  40 – 49  50 – 59  60 and above |
| --- | --- |
| Has a health professional ever told you that you have diabetes? | Yes – Type 1 diabetes  Yes – Type 2 diabetes  Yes – Gestational diabetes (diabetes during pregnancy)  Yes – Pre-diabetes  Yes – Diabetes but unsure what type  No  Prefer not to answer |

**Part 2 of 10**

**The questions below relate to your understanding about DNA and type 2 diabetes. Please rate how much you agree or disagree with each of the statements below:**

| I understand what DNA is. | Strongly disagree  Disagree  Agree  Strongly agree  Not sure |
| --- | --- |
| I understand how DNA is shared in families. | Strongly disagree  Disagree  Agree  Strongly agree  Not sure |
| I know that DNA can affect health. | Strongly disagree  Disagree  Agree  Strongly agree  Not sure |
| I know that DNA can affect disease. | Strongly disagree  Disagree  Agree  Strongly agree  Not sure |
| I think that changes in the DNA are linked to whether a person will get type 2 diabetes. | Strongly disagree  Disagree  Agree  Strongly agree  Not sure |
| Which of these sources of health advice do you trust? (You can select more than one answer) | GP, family doctor or nurse  Religious leader (e.g. Imam or Sheikh)  Alternative doctor (e.g. herbal practitioner,  homeopathic doctor or Ayurvedic doctor)  Friends  Family  Counsellor  Teacher  Youtube videos  Social media (e.g. Facebook, Instagram or Twitter posts)  None of the above |

**Part 3 of 10**

**The question below relates to your views about your personal risk for developing type 2 diabetes. Please answer the statement below:**

| What do you think your risk of getting type 2 diabetes in your lifetime is? | Almost no risk  A slight risk  A moderate risk  A high risk |
| --- | --- |

**Part 4 of 10**

**Please read the following explainer carefully:**

| **Your DNA and genetics run in your family and bloodline. It can affect your risk of getting certain health conditions. There are now tests and tools that have been developed to estimate an individual's genetic risk for diseases such as type 2 diabetes:**  **These genetic tests can be done using a sample of your saliva (spit). They will be able to tell you whether you are at low or high genetic risk of developing type 2 diabetes in the future. Genetic information in your body cannot be changed, and therefore, your genetic risk will be fixed in your lifetime. There may be lifestyle changes you can make to potentially prevent or delay type 2 diabetes (e.g. diet and physical activity). However, your genetic risk may affect whether these lifestyle changes will work for you.** |
| --- |

**Based on this information, please answer the questions below, which are related to your interest in genetic tests:**

| How important is it for you to know if you have a genetic risk for type 2 diabetes? | Not at all important  Not important  Somewhat important  Extremely important |
| --- | --- |
| If you were offered a genetic test for type 2 diabetes for free, how likely is it that you would take the test? | Not likely at all  Not likely  Somewhat likely  Extremely likely |
| At what age would you like to learn if you have a genetic risk for type 2 diabetes? | I would not like to know if I have a genetic risk for type 2 diabetes  Below 16  16 – 29  30 – 39  40 – 49  50 – 59  60 and above |
| Have you ever signed up for a personal genetic ancestry test (e.g. 23andme, Ancestry.com)? | Yes  No  Do not know  Prefer not to answer |

**Part 5 of 10**

**The questions below relate to your views about the outcomes and consequences of genetic testing for type 2 diabetes. Please rate how much you agree or disagree with each of the statements below:**

| Finding out about my genetic risk for developing type 2 diabetes would help me make important decisions about my health. | Strongly disagree  Disagree  Agree  Strongly agree |
| --- | --- |
| Getting a genetic test would be a frightening or stressful experience for me. | Strongly disagree  Disagree  Agree  Strongly agree |
| Getting a genetic test would be a frightening or stressful experience for my family and/or loved ones. | Strongly disagree  Disagree  Agree  Strongly agree |
| If a genetic test tells me that I have an above-average risk for type 2 diabetes, I think that health professionals can help support my health and well-being. | Strongly disagree  Disagree  Agree  Strongly agree |
| Finding out about my genetic risk for developing type 2 diabetes would be important for future generations in my family. | Strongly disagree  Disagree  Agree  Strongly agree |
| If a genetic test tells me that I have an above-average risk for type 2 diabetes, I am likely to experience fear, anxiety and/or depression. | Strongly disagree  Disagree  Agree  Strongly agree |

**Part 6 of 10**

**The questions below relate to your beliefs about the level of control you have over developing type 2 diabetes. Please rate how much you agree or disagree with each of the statements below:**

| Overall, I feel that I am able to control my health. | Strongly disagree  Disagree  Agree  Strongly agree |
| --- | --- |
| If I am going to get type 2 diabetes, I think that there is not much I can do about it. | Strongly disagree  Disagree  Agree  Strongly agree |
| I think that my personal health behaviours, such as diet and physical activity, can control my risks of getting type 2 diabetes. | Strongly disagree  Disagree  Agree  Strongly agree |
| If a genetic test tells me that I have an above-average risk for type 2 diabetes, then I would think that type 2 diabetes cannot be prevented. | Strongly disagree  Disagree  Agree  Strongly agree |

**Part 7 of 10**

**The question below relates to whether or not you have ever had a family member and/or close social contact diagnosed with type 2 diabetes. Please answer below:**

| In the people you have close social contact with in your life, do any of them have type 2 diabetes? (You can select more than one answer): | ☐ Mother  ☐ Father  ☐ Brother or sister  ☐ Any grandparents  ☐ Any aunts or uncles  ☐ Any cousins  ☐ Partner or spouse  ☐ Friend  ☐ Other – blood related  ☐ Other – not blood related  ☐ Prefer not to answer |
| --- | --- |

**Part 8 of 10**

**The questions below relate to the health behaviours of people living in your household. Please select your answers based on each of the statements below:**

| Including yourself, how many people are living together in your household (include those who usually live in the house such as students living away from home during term)? | Enter number: _____  Do not know  Prefer not to answer |
| --- | --- |
| People in my household work out, exercise, or participate in physical activity. | ☐ Never or very rarely  ☐ Once a week or less often  ☐ 2 – 4 times a week  ☐ 5 – 6 times a week  ☐ 1 – 2 times a day  ☐ 3 or more times a day  *Please note that physical activity is defined here as exercise carried out beyond daily work, for example running, going to the gym, walking, yoga.* |
| People in my household eat vegetables. Include fresh, tinned and frozen vegetables and pulses like lentils, chickpeas and kidney beans. | ☐ Never or very rarely  ☐ Once a week or less often  ☐ 2 – 4 times a week  ☐ 5 – 6 times a week  ☐ 1 – 2 times a day  ☐ 3 or more times a day |
| People in my household eat fruits. Include fresh, frozen, tinned and dried fruit. Do NOT count fruit juices. | ☐ Never  ☐ Less than half the time  ☐ About half the time  ☐ Most of the time  ☐ All of the time |
| People in my household cook with any of the following:   - butter - ghee - lard - coconut oil - palm oil | ☐ Never or very rarely  ☐ Once a week or less often  ☐ 2 – 4 times a week  ☐ 5 – 6 times a week  ☐ 1 – 2 times a day  ☐ 3 or more times a day |
| People in my household eat sugary foods such as:   - gulab jamun - mishti - halva - jalebi - rasmalai - sweets - biscuits - chocolate - cakes or cake rusks - sweet popcorn | ☐ Never or very rarely  ☐ Once a week or less often  ☐ 2 – 4 times a week  ☐ 5 – 6 times a week  ☐ 1 – 2 times a day  ☐ 3 or more times a day |
| People in my household drink sugary drinks such as:   - hot drinks with sugar (such as tea or coffee with sugar) - non-diet fizzy drinks - squashes - mixers - energy drinks - fruit juices - sweetened milk drinks - flavoured syrups | ☐ Never or very rarely  ☐ Once a week or less often  ☐ 2 – 4 times a week  ☐ 5 – 6 times a week  ☐ 1 – 2 times a day  ☐ 3 or more times a day |
| People in my household ask for snacks between meals such as:   - biscuits - chocolate - cakes - crisps - corn puffs - salted nuts - Bombay mix | ☐ Never or very rarely  ☐ Once a week or less often  ☐ 2 – 4 times a week  ☐ 5 – 6 times a week  ☐ 1 – 2 times a day  ☐ 3 or more times a day |
| When people in my household ask for unhealthy foods, other family members try to offer a healthy alternative. | ☐ Never  ☐ Less than half the time  ☐ About half the time  ☐ Most of the time  ☐ All of the time |

**Part 9 of 10**

**The questions below relate what you would do if a genetic test tells you that you have an above-average risk for type 2 diabetes. Please rate your answers based on each of the statements below:**

| If a genetic test tells me that I have an above-average risk for type 2 diabetes, I am likely to seek advice to prevent or delay type 2 diabetes. | ☐ Not likely at all  ☐ Not likely  ☐ Somewhat likely  ☐ Extremely likely |
| --- | --- |
| If a genetic test tells me that I have an above-average risk for type 2 diabetes, I am likely to seek advice from these sources (You can select more than one answer): | ☐ GP, family doctor or nurse  Religious leader (e.g. Imam or Sheikh)  Alternative doctor (e.g. herbal practitioner,  homeopathic doctor or Ayurvedic doctor)  Friends  Family  Counsellor  Teacher  Youtube videos  Social media (e.g. Facebook, Instagram or Twitter posts)  None of the above |
| If a genetic test tells me that I have an above-average risk for type 2 diabetes, I am likely to increase my physical activity to prevent or delay type 2 diabetes. | ☐ Not likely at all  ☐ Not likely  ☐ Somewhat likely  ☐ Extremely likely  *Please note that physical activity is defined here as exercise carried out beyond daily work, for example running, going to the gym, walking, yoga.* |
| If a genetic test tells me that I have an above-average risk for type 2 diabetes, I am likely to improve my dietary habits to prevent or delay type 2 diabetes. | ☐ Not likely at all  ☐ Not likely  ☐ Somewhat likely  ☐ Extremely likely |
| At the end of this survey, would you like to receive an email about some further online resources on health behaviours that can prevent or delay type 2 diabetes? | ☐ Yes  ☐ No |

**Part 10 of 10**

**There can be inequalities in type 2 diabetes risk and treatment according to the backgrounds**

**that people come from. We would like to ask you about your household income and**

**educational background so that we can understand these inequalities. For example, your**

**education may affect how well you understand information about DNA or type 2 diabetes, and**

**your income might affect whether you can follow a healthy diet or exercise programme.**

**These questions might be sensitive. Your responses will be stored securely and anonymously.**

**Any information you give us will remain confidential and it will only be used for the purposes**

**of this survey:**

| What is the average total income (before tax) received by your household? | Less than £18,000  £18,000 to £30,999  £31,000 to £51,999  £52,000 to £100,000  Greater than £100,000  Do not know  Prefer not to answer |
| --- | --- |
| Which of the following qualifications do you have? (You can select more than one answer): | College or University degree  A levels/AS levels or equivalent  O levels/GCSEs or equivalent  CSEs or equivalent  NVQ or HND or HNC or equivalent  Other professional qualifications: e.g. nursing, teaching  None of the above  Prefer not to answer |

References

1. Banerjee, A.T., et al., *Impact of the South Asian Adolescent Diabetes Awareness Program (SAADAP) on diabetes knowledge, risk perception and health behaviour.* HEALTH EDUCATION JOURNAL, 2022.

2. Gallagher, P., et al., *Patient beliefs and behaviors about genomic risk for type 2 diabetes: Implications for prevention.* Journal of Health Communication, 2015. **20**(6): p. 728-735.

3. Cameron, L.D., et al., *Impact of genetic risk information and type of disease on perceived risk, anticipated affect, and expected consequences of genetic tests.* Health psychology : official journal of the Division of Health Psychology, American Psychological Association, 2009. **28**(3): p. 307-16.

4. Patel, N.R., *The role of illness beliefs and social networks in South Asian people: A mixed-methods study*. 2012.

5. Patel, N.R., et al., *Illness beliefs and the sociocultural context of diabetes self-management in British South Asians: a mixed methods study.* BMC Fam Pract, 2015. **16**: p. 58.

6. van Esch, S.C.M., et al., *Family communication as strategy in diabetes prevention: an observational study in families with Dutch and Surinamese South-Asian ancestry.* Patient education and counseling, 2012. **87**(1): p. 23-9.

7. England, C.Y., et al., *Development of a brief, reliable and valid diet assessment tool for impaired glucose tolerance and diabetes: the UK Diabetes and Diet Questionnaire.* Public Health Nutrition, 2017. **20**(2): p. 191-199.

8. Emadian, A., C.Y. England, and J.L. Thompson, *Dietary intake and factors influencing eating behaviours in overweight and obese South Asian men living in the UK: mixed method study.* BMJ Open, 2017. **7**(7): p. e016919.

9. Seaborn, C., et al., *Utilizing Genomics through Family Health History with the Theory of Planned Behavior: Prediction of Type 2 Diabetes Risk Factors and Preventive Behavior in an African American Population in Florida.* PUBLIC HEALTH GENOMICS, 2016. **19**(2): p. 69-80.
