# Supplementary material for: Perceived risk of type 2 diabetes: Using linked genomic, clinical and questionnaire data to understand the potential use of genetic risk tools in British South Asians
Source: PLOS Glob Public Health. 2025 Mar 31;5(3):e0004274. doi: 10.1371/journal.pgph.0004274 (PMC11957276; doi:10.1371/journal.pgph.0004274)
Supplement: S4 Appendix — (DOCX) [file pgph.0004274.s004.docx]

S4 Appendix. Comorbidities included in analysis.

Number of comorbidities in this study was defined as an index reflecting accumulation across the following conditions for each participant—guided by the NHS Quality and Outcomes Framework clinical and public health indicators for 2023/24 in England (listed in alphabetical order):

1. Anxiety
2. Asthma
3. Atrial fibrillation
4. Bipolar affective disorder and mania
5. Cancer
6. Chronic kidney disease
7. Chronic obstructive pulmonary disease
8. Coronary heart disease
9. Dementia
10. Depression
11. Epilepsy
12. Heart failure
13. Hypertension
14. Learning disabilities
15. Obesity
16. Osteoporosis
17. Peripheral arterial disease
18. Polycystic ovary syndrome *
19. Rheumatoid arthritis
20. Schizophrenia and other psychoses
21. Smoking status
22. Stroke

* Polycystic ovary syndrome is not an NHS Quality and Outcomes Framework clinical or public health indicator. However, it was included in this list, given its role as a known significant risk factor for T2D.
