# Supplementary material for: Perceived risk of type 2 diabetes: Using linked genomic, clinical and questionnaire data to understand the potential use of genetic risk tools in British South Asians
Source: PLOS Glob Public Health. 2025 Mar 31;5(3):e0004274. doi: 10.1371/journal.pgph.0004274 (PMC11957276; doi:10.1371/journal.pgph.0004274)
Supplement: S5 Appendix — (DOCX) [file pgph.0004274.s005.docx]

S5 Appendix. Comparison of genomic and clinical data between responders and non-responders.

To account for possible selection bias in our study sample, we compared relevant genomic and clinical characteristics between three different groups of survey response status from the pool of Genes & Health volunteers to whom we sent out invitations—1) volunteers who completed our questionnaire; 2) volunteers who began answering the questionnaire but did not complete it and 3) volunteers who did not respond to our invitations. As shown in the table below, the characteristics of these different groups of individuals did not vary significantly—at least in terms of their T2D PRSs, BMI and number of comorbidities (S1 Table).

| Mean (SD) | | | |
| --- | --- | --- | --- |
|  | **T2D PRSs** | **BMI** | **Number of comorbidities** |
| Complete | – 0.13 (1.00) | 25.9 (4.95) | 1.42 (1.36) |
| Incomplete | – 0.09 (0.95) | 26.9 (4.76) | 1.25 (1.24) |
| Non-response | – 0.11 (1.00) | 26.4 (4.89) | 1.43 (1.21) |

S1 Table. Comparison of genomic and clinical data between three different groups of survey response status.

One-way ANOVAs confirmed that these differences were not statistically significant—with *F*(2, 4367) = 0.11, *p* = 0.89; *F*(2, 3455) = 1.90, *p* = 0.15 and *F*(2, 4952) = 0.96, *p* = 0.38 for T2D PRSs, BMI and number of comorbidities, respectively.
