# Supplementary material for: Perceived risk of type 2 diabetes: Using linked genomic, clinical and questionnaire data to understand the potential use of genetic risk tools in British South Asians
Source: PLOS Glob Public Health. 2025 Mar 31;5(3):e0004274. doi: 10.1371/journal.pgph.0004274 (PMC11957276; doi:10.1371/journal.pgph.0004274)
Supplement: S6 Appendix — (DOCX) [file pgph.0004274.s006.docx]

S6 Appendix. Results from confirmatory factor analysis.

Confirmatory factor analysis was conducted to assess the validity of the latent variables included in our model. Cronbach’s alpha coefficients were also obtained to evaluate measurement reliability for these constructs. An initial measurement model demonstrated acceptable fit, χ^2^(362) = 1680.75, *p* < 0.05, CFI = 0.77, TLI = 0.74 and RMSEA = 0.08, 90% CI [0.07, 0.08] (S2 Fig). Curved double-headed arrows in the figure represent the variance of a variable—or covariance between two variables.


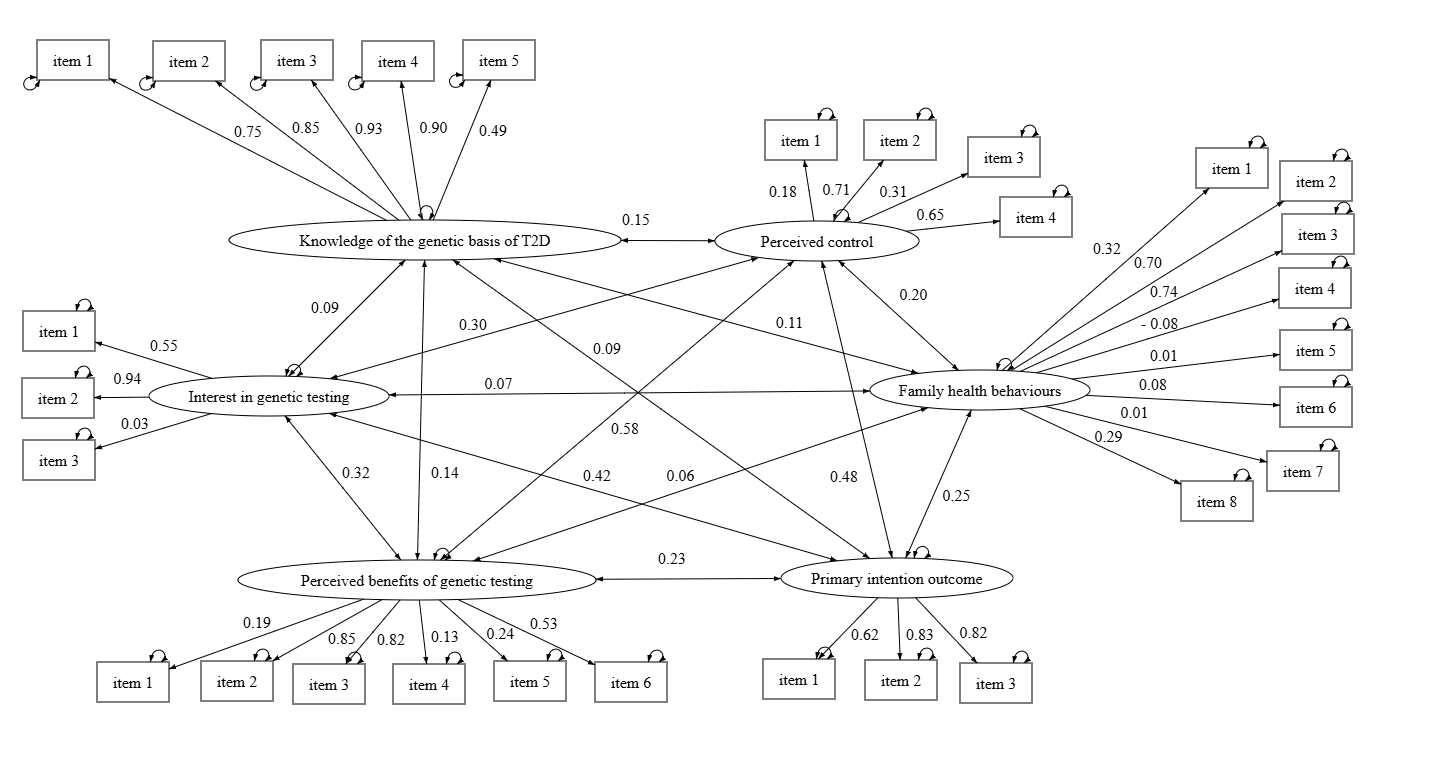


S2 Fig. Initial measurement model for latent variables.

For knowledge of the genetic basis of T2D, factor loadings were significant for all five items on the construct. Cronbach’s alpha coefficient was 0.89, suggesting good internal consistency. The measure was thus retained for analysis in the full model.

Interest in genetic testing was reduced into a one-item construct based on the item in the questionnaire with the highest factor loading and also deemed most theoretically relevant to the current study—“If you were offered a genetic test for type 2 diabetes for free, how likely is it that you would take the test?”. This was included in the final model as an observed variable.

For both perceived control and perceived benefits of genetic testing, factor loadings varied considerably within each construct—and internal consistency ranged from a poor 0.52 to 0.66. Due to some overlap between the content of questions in these two constructs, exploratory factor analysis was applied to a subset of the questionnaire data to review the dimensionality of these two sets of questions. Fitting a one-factor model showed that five items across these measures had factor loadings of over 0.30 (S2 Table). These items were mostly related to fear and anxiety over potentially negative outcomes of genetic testing—particularly a sense of loss of control over T2D prevention (S2 Table). A newly defined measurement model—combining this subset of five items—was then tested for a single measure of perceived control to be used in place of the two original measures. Cronbach’s alpha coefficient was 0.77 for this new measure, indicating improved internal consistency.

|  | Factor loading | Communality |
| --- | --- | --- |
| Getting a genetic test would be a frightening or stressful experience for me. | 0.81 | 0.65 |
| Getting a genetic test would be a frightening or stressful experience for my family and/or loved ones. | 0.76 | 0.58 |
| If a genetic test tells me that I have an above-average risk for type 2 diabetes, I am likely to experience fear, anxiety and/or depression. | 0.58 | 0.34 |
| If I am going to get type 2 diabetes, I think that there is not much I can do about it. | 0.48 | 0.23 |
| If a genetic test tells me that I have an above-average risk for type 2 diabetes, then I would think that type 2 diabetes cannot be prevented. | 0.55 | 0.30 |

S2 Table. Results from the exploratory factor analysis for perceived control and perceived benefits of genetic testing.

For the originally defined family health behaviours construct, factor loadings also varied considerably—and internal consistency was a poor 0.54. Exploratory factor analysis was similarly applied to a subset of the questionnaire data to review the dimensionality of this construct. Fitting a one-factor structure showed that four items relating to dietary habits in the family environment had factor loadings of over 0.30. Cronbach’s alpha coefficient for this four-item measure was, however, still unsatisfactory—at 0.58—and item statistics indicated that dropping the final item on the measure would improve reliability. Only three items were therefore included in the newly defined measurement of family health behaviours for the final model (S3 Table). Cronbach’s alpha coefficient was 0.70, suggesting good internal consistency.

|  | Factor loading | Communality |
| --- | --- | --- |
| People in my household eat sugary foods such as:   - gulab jamun - mishti - halva - jalebi - rasmalai - sweets - biscuits - chocolate - cakes or cake rusks - sweet popcorn | 0.60 | 0.36 |
| People in my household drink sugary drinks such as:   - hot drinks with sugar (such as tea or coffee with sugar) - non-diet fizzy drinks - squashes - mixers - energy drinks - fruit juices - sweetened milk drinks - flavoured syrups | 0.63 | 0.40 |
| People in my household ask for snacks between meals such as:   - biscuits - chocolate cakes - crisps - corn puffs - salted nuts - Bombay mix | 0.72 | 0.51 |

S3 Table. Results from the exploratory factor analysis for family health behaviours.

For the primary intention measure, factor loadings were significant for all three items on the construct. Cronbach’s alpha coefficient was 0.79, suggesting good internal consistency. The three-item measure was therefore retained for interpretation in the final model.

A revised model incorporating all the above changes was tested—and this demonstrated a slightly better fit than the initial model, χ^2^(98) = 254.54, *p* < 0.05, CFI = 0.92, TLI = 0.91 and RMSEA = 0.07, 90% CI [0.06, 0.08]. Modification indices were subsequently examined to identify further improvements—and these suggested the inclusion of correlated uniqueness between some of the items on the knowledge of the genetic basis of T2D scale, as well as on the newly defined measure of perceived control. The final measurement model showed a good fit, χ^2^(95) = 166.46, *p* < 0.05, CFI = 0.98, TLI = 0.98 and RMSEA = 0.04, 90% CI [0.03, 0.04]. This is presented in S3 Fig below, alongside all standardised factor loadings.


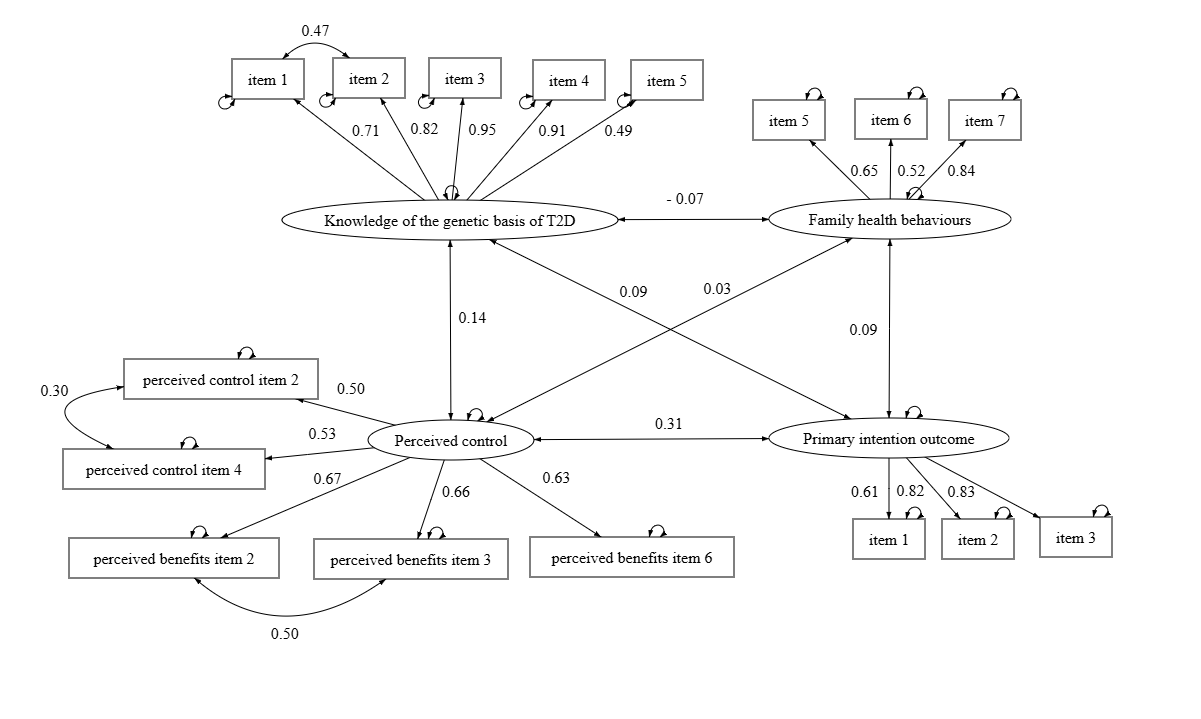


S3 Fig. Final measurement model for latent variables.
