# Supplementary material for: Perceived risk of type 2 diabetes: Using linked genomic, clinical and questionnaire data to understand the potential use of genetic risk tools in British South Asians
Source: PLOS Glob Public Health. 2025 Mar 31;5(3):e0004274. doi: 10.1371/journal.pgph.0004274 (PMC11957276; doi:10.1371/journal.pgph.0004274)
Supplement: S7 Appendix — (DOCX) [file pgph.0004274.s007.docx]

S7 Appendix. Imputation diagnostics.

Multiple imputation by predictive mean matching was applied alongside our SEM analysis to create and analyse 30 imputed datasets—estimating missing values for participants’ T2D PRSs and BMI, based on all other variables included in our hypothesised model (e.g. age, sex and family history of T2D). Imputation diagnostics were then performed to inspect our imputed data. Firstly, descriptive statistics of the observed and imputed values are presented in S4 Table below. Next, the distribution of these observed and imputed values for T2D PRSs and BMI are compared in S4 Fig and S5 Fig, respectively. Finally, S6 Fig shows traceplots of the means and SDs of imputed values against the iterations of imputed data—the lack of distinctive trends in the parallel streams demonstrate convergence of the imputation algorithm.

|  | Observed values | | | Imputed values | | |
| --- | --- | --- | --- | --- | --- | --- |
|  | Minimum | Mean | Maximum | Minimum | Mean | Maximum |
| T2D PRSs | – 3.39 | – 0.13 | 2.68 | – 3.39 | – 0.14 | 2.68 |
| BMI | 13.33 | 25.92 | 46.70 | 13.33 | 25.42 | 46.70 |

S4 Table. Descriptive statistics of observed and imputed values for T2D PRSs and BMI.


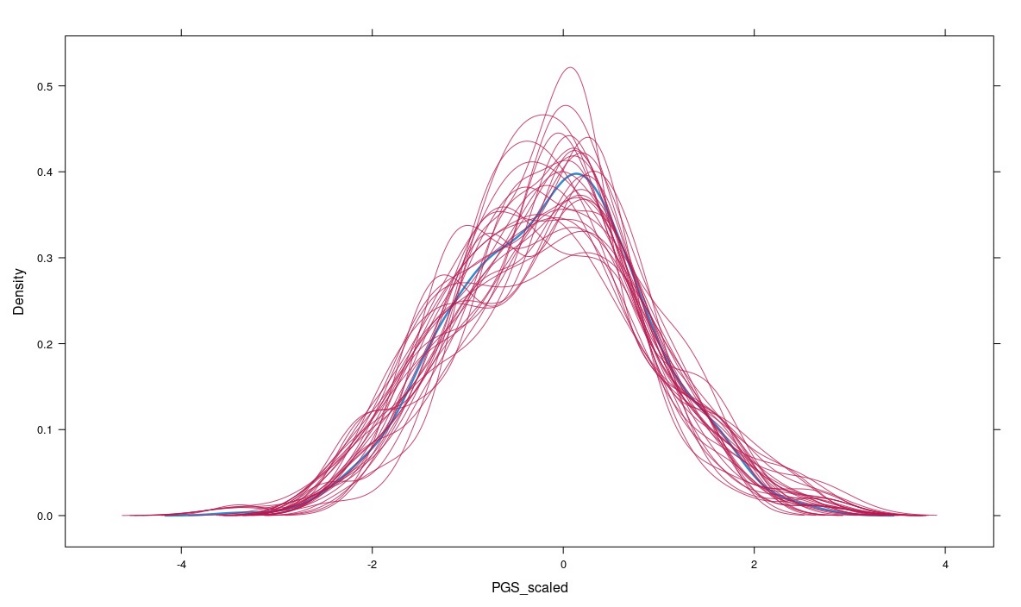


S4 Fig. Distribution of observed (blue) and imputed (red) values for T2D PRSs.


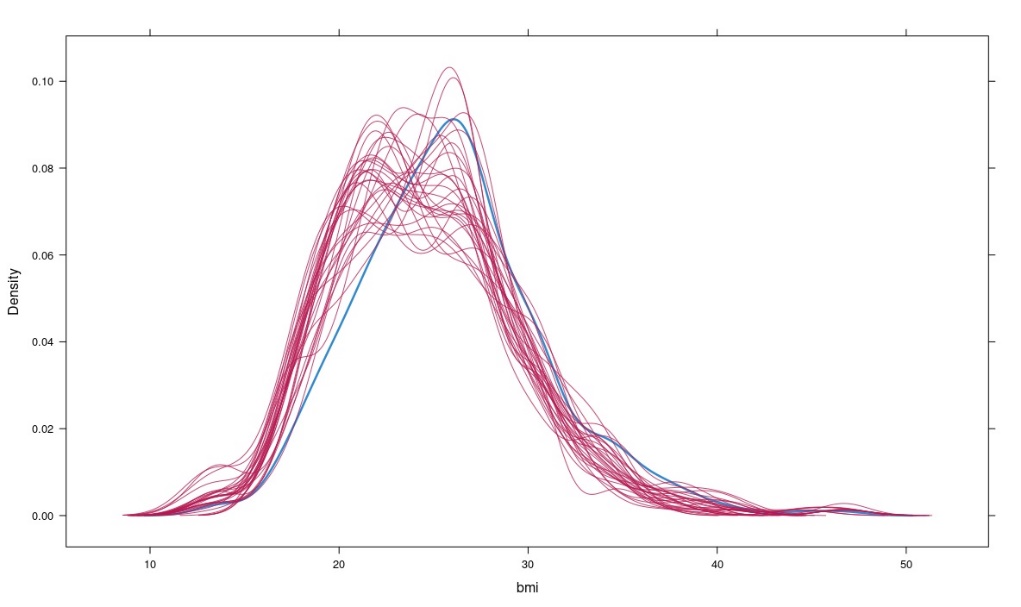


S5 Fig. Distribution of observed (blue) and imputed (red) values for BMI.


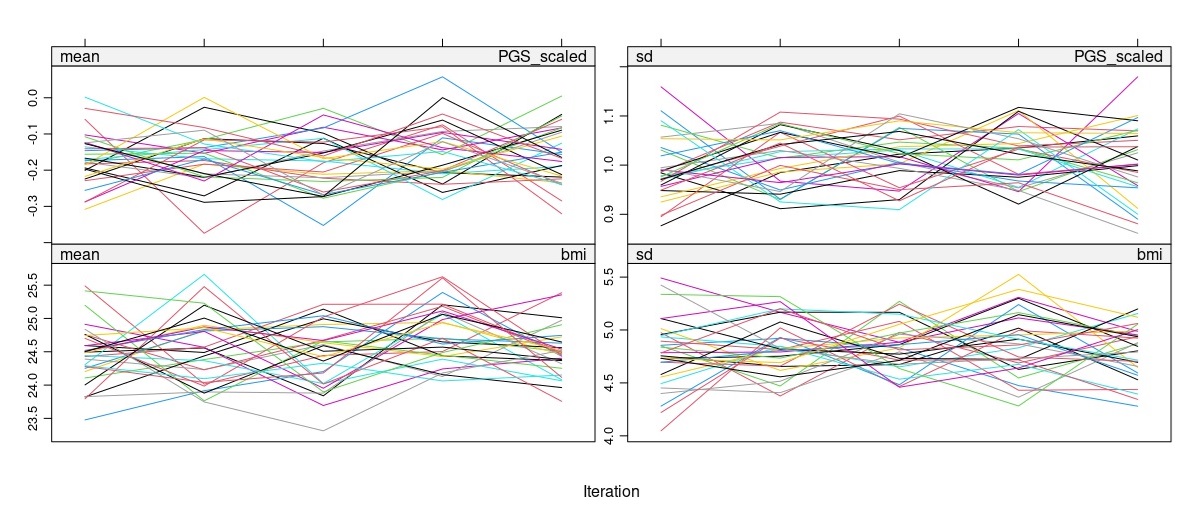


S6 Fig. Traceplots demonstrating convergence of the imputation algorithm for T2D PRSs and BMI.
