# Supplementary material for: Perceived risk of type 2 diabetes: Using linked genomic, clinical and questionnaire data to understand the potential use of genetic risk tools in British South Asians
Source: PLOS Glob Public Health. 2025 Mar 31;5(3):e0004274. doi: 10.1371/journal.pgph.0004274 (PMC11957276; doi:10.1371/journal.pgph.0004274)
Supplement: Attachment — Submitted filename: response_letter_auresp_3.docx [file pgph.0004274.s012.docx]

Dear editor and reviewer,

Thank you for your time spent on our manuscript entitled “*Perceived risk of type 2 diabetes: Using linked genomic, clinical and questionnaire data to understand the potential use of genetic risk tools in British South Asians*”.

We are pleased to see that all comments previously raised by Reviewer #2 has been satisfactorily addressed. We thank them again for their valuable feedback, which has helped us refine our manuscript further. We are grateful for their appreciation of our study as an important addition to the literature.

A point-by-point response to the additional comments raised can be found below:

***Reviewer comments***

*Materials and methods:*

*SEM section:*

*Paragraph 1: “The main exogenous variable included in our hypothesised model as shown in Fig 2 was a binary variable for age—according to the age group with the largest n in our sample—to aid interpretation (46 – 59 years = 1; all other age groups = 0).*

- *How does this binary encoding of the age variable aid in interpretation? Why was this encoding chosen, in particular because Table 2 (participant characteristics) specifies four separate age groups of various sizes?*
- *What are limitations of this choice?*

We thank the reviewer for this reflection. In our view, converting the age variable into a binary factor was a more straightforward choice than including all four age groups—as the latter approach would necessitate more complex multi-group analysis in SEM. These would essentially test out separate models across four discrete groups (according to the four age groups defined in our questionnaire), which would consequently result in four models to interpret. This, in our opinion, is neither efficient nor useful.

The oldest age group in our sample (46 to 59 years) was chosen as our indicator variable, as it was the age group with the largest *n* (this decision is specified in p. 9 of our manuscript). We appreciate that depth may be lost following the reduction of multiple age groups into two categories—as well as the loss of the ability to explore specific group differences—but we felt it necessary to aid with a more direct interpretation of a singular model.

*Paragraph 3: ”The inclusion of both T2D PRSs and family history of T2D in our model may also introduce confounding bias—however, it was in our interest to examine the potential mediating relationships between genetic and familial risk of T2D, in light of emerging evidence on their independent and complementary effects on inherited disease susceptibility.”*

- *Would split this into two shorter statements and repeat exact phrasing for clarity: “The inclusion of both T2D PRSs and family history of T2D in our model may introduce confounding bias. However, in light of emerging evidence of their independent and complementary effects on T2D susceptibility, we examined the potential mediating relationships between T2D PRSs and family history of T2D.”*

We thank the reviewer for this suggestion. This wording has now been updated in the manuscript (p. 10).

*Discussion:*

*Paragraph 5:*

*“Interventions can also be supplemented with further system-level services—incorporating elements such as environmental restructuring or social planning—to facilitate the translation from risk awareness into actual preventative action across diverse groups.”*

- *Would break this into two separate sentences, omitting the dash, and provide examples of such systems-level services and cite examples.*

This is now revised on p. 14 of the manuscript.

*Paragraph 7:*

*Very long sentence, again with unnecessary use of dash: “The inclusion of both T2D PRSs and family history of T2D in our SEM analysis may also introduce confounding bias—however, as mentioned in our methods section, it was in our interest to examine the potential mediating relationships between genetic and familial risk of T2D, in light of emerging evidence on their independent and complementary effects on inherited disease susceptibility.”*

- *Would break into 2 sentences and rephrase as in the SEM section: “The inclusion of both T2D PRSs and family history in our SEM analysis may have introduced confounding bias. However, as discussed in the methods section, we examined the potential mediating relationships between genetic risk of and family history of T2D.”*

This section on p. 15 has now been updated to reflect earlier wording in the SEM analysis section.

*Paragraph 8:*

*Would clean up this sentence: “In future work, it may be that even seemingly objective sociodemographic measures also need to undergo a process of tailoring and refining to ensure that items are capturing the right categories in a diverse sample.”*

- *What tailoring and refining do you refer to? The previous sentences speak about representativeness of the study population; perhaps this sentence could discuss that more broadly as well.*

Here, we are referring to the fact that the educational and income categories we used in our questionnaire (i.e. those previously defined in UK Biobank) might not necessarily reflect the same groups in Genes & Health. For educational level, for example, there may be other factors to consider, such as our participants’ immigration status, the countries where their educational qualifications were obtained—and whether such qualifications are equivalent to traditional definitions of being college- or university-educated in the UK. The idea of “tailoring and refining” relates to how future work can further define categories that can most accurately capture seemingly “objective” socio-demographic measures such as educational level and household income. We have refined our wording on p. 15 for clarity.

*Paragraph 9:*

*Last line is a very long sentence: “It will also be important to consider how these strategies can be tailored to be applicable to younger age groups—given the discordance found in this study between the age at which individuals would want to find out about their genetic risk of T2D, versus the age at which they would actually be ready, or willing, to implement preventative lifestyle changes.”*

- *Would split into two and clarify: “In the present study, we found that there was a discordance between the age group at which individuals would want to learn about their genetic risk of T2D versus the age at which they would be ready and willing to implement preventative lifestyle changes. It will be important to consider how the integration of genomics into heath systems can be tailored to younger populations.”*

This is now revised on p. 16 of the manuscript.

We hope to have addressed all questions and concerns raised by the reviewer. Thank you again for your time and consideration.

Kind regards,

Law et al.
