# Supplementary material for: Perceived risk of type 2 diabetes: Using linked genomic, clinical and questionnaire data to understand the potential use of genetic risk tools in British South Asians
Source: PLOS Glob Public Health. 2025 Mar 31;5(3):e0004274. doi: 10.1371/journal.pgph.0004274 (PMC11957276; doi:10.1371/journal.pgph.0004274)
Supplement: Attachment — Submitted filename: responses_reviewers_comments.docx [file pgph.0004274.s009.docx]

Dear editor and reviewers,

Thank you for your time spent on our manuscript entitled “Perceived risk of type 2 diabetes: Using linked genomic, clinical and questionnaire data to understand the potential use of genetic risk tools in British South Asians”.

The authors would first like to thank all three reviewers for their very detailed and constructive feedback on our manuscript. We are particularly grateful for their appreciation of the novelty of our study in integrating various data resources to examine genetic risk perceptions surrounding T2D. This work is particularly important in Bangladeshi and Pakistani populations so disproportionately affected by the condition—yet so underrepresented in existing research.

We have addressed all comments and suggestions raised by the reviewers in our revised manuscript (please see the tracked version of our submission to view specific changes). Some of the major changes we have made include: 1) moving information about our CFA analysis into the main text, as well as 2) describing further detail about model specification in our methods section. Additionally, we have included a comparison of relevant genomic and clinical characteristics across our questionnaire responders and non-responders in the appendix—in order to address reviewer concerns surrounding selection bias. A point-by-point response to these comments can be found below:

***Reviewer #1***

*Thanks for the opportunity to read your manuscript. My role is statistical reviewer, so I have focused on the design, data, and analysis that are presented. I have put general comments first, followed by questions relevant to a specific section of the manuscript (with a page/line reference).*

*This study examines attitudes to genetic risk of T2DM in British people of Pakistani and Bangladeshi decent. Data comes from a large study (>50k) specifically recruiting people from this population to provide samples to a biobank. This is a sub-study, which approached participants in the main study to provide self-report information on genetic risk perception. The study included participants 16-59 without DM and were able to be contacted for participation. Stratified (by age-group) quota sampling was used to achieve a sample of 626 participants (response rate of 17%). Participants reported genetic risk perception, family history of T2D, and questions about intentions for preventative behaviour and receiving preventative information. Participants genetic risk for T2DM was estimated previously published risk prediction tools. Links to clinical data were used to access BMI and comorbidities. CFA was used to construct latent variables from self-report information, and a revised model reducing items showed adequate fit indices. Imputation with MICE was used to deal with missing data in the study. The main analysis used DWLSE, model fit for the main model was good. Mediation effects were estimated with MC from data generated from the main estimates.*

1. *The approach to the main analysis (measurement model, refined measurement model, main model) is reasonable and the model fit is good. There was very little provided about the process to define the relationships between the study variables in the main SEM. This is an area where there are theoretical models that exist (e.g. Theory* *of Planned Behaviour) but it's not clear to me how the main model was conceived. This is important detail that needs to be included.*

We thank the reviewer for this reflection. The process of defining and testing hypothesised relationships between different variables in SEM—i.e. model specification—is indeed often grounded in existing theories or frameworks. In the present study, our model builds on a diverse body of work in the literature on genetic risk perceptions and health behaviours. Naturally, it draws from prominent theories in the research area, including (but not limited to) the Theory of Planned Behaviour, as referenced by the reviewer—a framework specifying the intention-behaviour relationship. The Health Belief Model is another model that we have drawn from—and it integrates other important factors that have been shown to influence health behaviours, such as perceived benefits and perceived barriers. These theories have been heavily used by researchers as guiding frameworks in attempts to chart the prediction of health behaviours based on constructs such as perceived risk, perceived control, intention etc.—all of which we have similarly included in our study. However, we would also like to acknowledge that theory alone often lacks the requisite detail to provide a comprehensive guide for model specification in SEM. In our case, given the lack of understanding surrounding genetic risk perceptions in a sample of our characteristics in the wider literature, it is likely that existing models/theories may not be sufficient to allow for a full understanding of health-related perceptions and beliefs in British Bangladeshi and British Pakistani populations. For this reason, we have adapted a model specifying more complex relationships between these genetic risk perceptions—and further integrated external variables with plausible influence on individuals’ perceptions and behaviours, such as family history of T2D. The point of the main analysis is precisely to test these relationships, based on their theoretical plausibility for this sample. Thus, whilst building on existing theory, we have been able to chart out a more complex picture of genetic risk perceptions in this underrepresented sample—evaluating and appraising the fit of our model against these theoretical propositions. We have now integrated further explanation on the theoretical background and generation of our model in the manuscript (pp. 8 – 9).

2. *The response rate for this specific study was low (17%), after accounting for the sampling approach was the sample for this study similar to the main study? i.e. does it look as though non-response was random, and without selection on colliders?*

We acknowledge the low recruitment rate in our study as a potential limitation, especially in terms of generalisability—as this may introduce bias to our study findings. However, firstly, we would like to argue that our recruitment rate is relatively comparable to figures that have been reported for online questionnaire studies that have been conducted in other large genomic cohorts such as FinnGen (p. 7). The authors recalled biobank participants to collect cognitive, behavioural and lifestyle information in 2021—and reported a response rate of 18.6%. In our case, we were unable to check and filter email addresses that were not in use in Genes & Health (approximately 26%)—and had to cold email participants without establishing prior contact for the study, thereby inflating the non-response rate. Given these circumstances, we were pleased to still achieve a sample size with a relatively balanced representation for our study. Additionally, we have conducted a comparison of the relevant genomic and clinical characteristics between responders and non-responders in our sample. This suggests that they do not differ significantly—at least in terms of their T2D PRSs, BMI and number of comorbidities. We have updated the manuscript (p. 7) and appendix (S4 Appendix; p. 12) to include this information.

3. *P7, Paragraph 3. Which methods within the MICE model were used for the different variables, e.g. PMM or linear regression? How was 30 imputations decided on?*

We conducted multiple imputations by PMM in our SEM analysis—and have now included this detail in the data analysis section of our manuscript (p. 9). We took an iterative approach to determine the number of imputations needed, increasing the number of imputed datasets in a stepwise manner until the estimates around T2D PRSs and BMI converged. Through these procedures, we relied on imputation diagnostics to compare our observed and imputed values. Details about these diagnostics are now included in S6 Appendix to demonstrate the validity of our imputations (pp. 15 – 16).

4. *P8, Paragraph 1. Information about the estimation for the SEM is probably better in the methods. Was diagonally weighted least squares used so that the self-report variables could be used as ordinal variables instead of assuming a Likert scale?*

We have now moved this information to the methods section of the manuscript and provided proper justification for the use of DWLS (p. 9).

5. *P8. Fig 2. The caption/title for this needs updating, with coefficients and significance indicators on paths this is no longer a hypothesised model.*

We haven now included a separate figure in our methods section to differentiate between our hypothesised (p. 7) and tested (p. 10) models in the manuscript. The caption for Fig 2 (now Fig 4; p. 10) has also been updated accordingly.

6. *P9, Paragraph 4. Is it possible to apportion the total effect into direct and indirect (mediated) effects?*

We reported only indirect effects in our results section for two reasons. Firstly, as our primary interests lie in testing possible mediating relationships between various socio-demographic, clinical and familial variables in informing participants’ genetic risk perceptions (pp. 3 – 4)—we make no hypothesis around direct effects between, e.g., age group and the secondary intention outcome, as well as between ethnicity and the secondary intention outcome (Fig 2; p. 7). Secondly, we conducted mediation analysis only on pathways with significant links to the outcome measures. In the case of ethnicity, for example, given all other pathways (involving BMI or comorbidities) showed no evidence of direct effects (Fig 4; p. 10), the total effects for these pathways could not be obtained.

***Reviewer #2***

*In this paper, the authors used structural equation modelling to explore genetic risk perceptions surrounding type 2 diabetes (T2D) in British, Bangladeshi and Pakistani volunteers. While their effort is laudable, the paper has several limitations.*

1. *The response rate is extremely low (17%). This can affect the results of the study as there could be referral bias in the sample.*

We thank the reviewer for raising this concern. We acknowledge the low recruitment rate in this study as a potential limitation, especially in terms of generalisability—as this may introduce bias to our study findings. However, firstly, we would like to argue that our recruitment rate is relatively comparable to figures that have been reported for online questionnaire studies that have been conducted in other large genomic cohorts such as FinnGen (p. 7). The authors recalled biobank participants to collect cognitive, behavioural and lifestyle information in 2021—and reported a response rate of 18.6%. In our case, we were unable to check and filter email addresses that were not in use in Genes & Health (approximately 26%)—and had to cold email participants without establishing prior contact for the study, thereby inflating the non-response rate. Given these circumstances, we were pleased to still achieve a sample size with a relatively balanced representation for our study. Additionally, we have conducted a comparison of the relevant genomic and clinical characteristics between responders and non-responders in our sample. This suggests that they do not differ significantly—at least in terms of their T2D PRSs, BMI and number of comorbidities. We have updated the manuscript (p. 7) and appendix (S4 Appendix; p. 12) to include this information.

2. *The age of the participants is from 46 to 59 years. In over 50% of South Asians, diabetes occurs below 45 years of age. Hence, this may not be representative of younger south Asians with type 2 diabetes where this study would be more relevant.*

We would like to clarify that our study sample does, in fact, include participants from younger age groups (this detail is described in p. 4 and p. 9 of the manuscript). In our main analysis, however, we have specified the 46 to 59 year age group as the indicator variable for age in our model (p. 8) simply because 1) they represented the largest subsample in our study (*n* = 236; 37.70%) and 2) descriptive statistics showed that participants in this oldest age group reported the highest levels of intention to adopt health behaviours. As our main analysis was concerned predominantly with identifying the characteristics of individuals who are likely to act on genetic risk information about T2D, it made sense for us to place focus on the age group reporting the highest levels of intention to aid interpretation—i.e. in terms of drawing comparisons between participants in this age group vs. all other groups. We have, however, emphasised the benefits of PRSs in detecting T2D risk in younger populations in our manuscript (p. 13), echoing the concerns of the reviewer, alongside acknowledging the complexity of the relationships between age and readiness to engage in health behaviours (pp. 12 – 13).

3. *There is female bias as 62.6% of the participants were female.*

We acknowledge the possibility of gender bias in our sample, which is related to the moderate oversampling of female participants in Genes & Health. However, as described in the cohort profile, the Genes & Health sample remains broadly representative of the background population it aims to represent, with regard to demographic characteristics such as age and sex (<https://academic.oup.com/ije/article/49/1/20/5555939>). We have nonetheless acknowledged this as a possible limitation in our discussion section (p. 13).

4. *There could be other biases as well due to the study design. It is therefore not clear whether the findings observed are applicable to all south Asians with diabetes in the UK.*

We would like to argue that although the current study is purely cross-sectional and observational in design—which definitely comes with inherent biases—it also has particular strengths in addressing an area of research that has remained largely uncovered by existing work in the literature. On one level, there has been historical underrepresentation of non-White populations in genomics research. This study addresses some of these gaps by providing novel and unique insights into genetic risk perceptions surrounding T2D in an underrepresented population so disproportionately affected by the condition. Additionally, we have been able to leverage different sources of data in Genes & Health to build a more comprehensive picture of wider influences that may be informing these genetic risk perceptions. We have emphasised these strengths in our discussion section (p. 14)—and we make no assumptions in regards to generalisability, highlighting that future research should build on this work to explore these issues more widely (p. 13).

***Reviewer #3***

*In this paper, the author randomly selected ~600 participants from ~50,000 participants with Pakistan and Bangladesh descent to participate an online survey to explore the inter-relationships amongst their knowledge about genetics of diabetes, their demographic and family information, perceived risk of diabetes, interest to know their genetic risk and readiness to take preventive actions. Using variables from a set of questionnaires designed to test this conceptual framework, they derived latent variables and used SEM to test their interrelationships. They reported that people of older age were more keen to learn about their genetic risk and take actions to reduce their risk of diabetes, including receiving email from NHS later on to support behavioural change. These participants were enrolled in the Genes and Health Consortium in whom PRS based on European data had been generated aimed at informing genetic risk and motivating behavioural changes.*

*In this SEM, only positive family history of diabetes directly influenced their perceived genetic risks which in turn influenced their interest to get tested and take preventive action. The interest to test was also influenced by the income level. The T2D PRS was upstream of family history, BMI and comorbidities while female appeared to have more comorbidities indicated by a path. Age indirectly influenced BMI and comorbidities which might have influence on perceived risk. While participants indicated that they preferred to learn about their genetic risk at an earlier age of ~ 30, it was only at a later age that they were ready to take preventive actions creating a misconnect between interest and action in these young people. The family health behaviour also influenced their knowledge, perceived risk and locus of control. The authors concluded that in order to combine clinical factors and PRS in clinical practice for identifying high risk people for preventive actions especially relevant to young people in whom genetic risk may be more informative than clinical risk, there are sociodemographic and contextual barriers that need to be overcome through a family and a system approach. In the appendix, the authors mentioned that having free genetic tests was the single most important determinant for having the test. Besides, only 17% of people responded to the questionnaire.*

*This is a timely article and is well thought out. Overall, the paper is easy to read and follow with interesting observations that are relevant to a large scale implementation of precision prevention or care in diabetes. However some points need to be clarified:*

1. *It is not clear whether participants of the Genes and Health Consortium were given their PRS for diabetes - this need to be stated explicitly.*

Thank you for drawing our attention to this potential source of confusion. We would like to clarify that participants in the study were not provided with their PRSs for T2D—thus were unaware of their genetic risk status for the condition. This detail is now emphasised in p. 5 of the manuscript.

2. *The low response rate is concerning, only 1 in 5 people responded to the survey - has this low participation rate already given us the answer and that only 1 in 5 were interested in responding to genetic-test related surveys. A comparison between the responders and non-responders will be very informative.*

We acknowledge the low recruitment rate in this study as a potential limitation, especially in terms of generalisability—as this may introduce bias to our study findings. However, firstly, we would like to argue that our recruitment rate is relatively comparable to figures that have been reported for online questionnaire studies that have been conducted in other large genomic cohorts such as FinnGen (p. 7). The authors recalled biobank participants to collect cognitive, behavioural and lifestyle information in 2021—and reported a response rate of 18.6%. In our case, we were unable to check and filter email addresses that were not in use in Genes & Health (approximately 26%)—and had to cold email participants without establishing prior contact for the study, thereby inflating the non-response rate. Given these circumstances, we were pleased to still achieve a sample size with a relatively balanced representation for our study. Additionally, we have conducted a comparison of the relevant genomic and clinical characteristics between responders and non-responders in our sample. This suggests that they do not differ significantly—at least in terms of their T2D PRSs, BMI and number of comorbidities. We have updated the manuscript (p. 7) and appendix (S4 Appendix; p. 12) to include this information.

3. *An original causal model should be presented with clear language about the positive and negative relationships amongst different variables. The factor loading onto various latent variables in the supplementary text should be presented followed by the final model in the main text.*

We haven now included a separate figure in our methods section to differentiate between our hypothesised (p. 7) and tested (p. 10) models in the manuscript. We have also integrated further detail in our explanation for using SEM as an approach, as well as more information about the theoretical basis for the generation of our proposed model (pp. 7 – 9). Discussions about the positive and negative relationships amongst different variables, however, are reserved for our results and discussion sections—as we make no assumptions about the directionality of these relationships at the model specification stage in our SEM analysis. Details around factor loadings have been integrated from the appendix to the main text (p. 8), as suggested by the reviewer.

4. *There should be clear explanatory note in these different models to help readers understand the concept and relationships amongst these variables which implies causal relationship. The final model lacks description in the legend. There is also no statistics to show the significance of the regression coefficients of different paths. The explained variance of these variables due to direct and mediating effects of different factors as well as the total variance explained by the causal model should be stated.*

In explaining the theoretical basis for the generation of our proposed model (pp. 7 – 9)—as well as including further information about our CFA analysis in the main text (p. 8)—we hope that we have now provided a bit more clarity on the concept and relationships between the variables included in our model. In regards to the significance of paths, it is standard to denote significant paths with solid lines and non-significant paths with dashed lines in SEM, which we have already clarified in p. 10 of the manuscript. We reported only indirect effects in our results section for two reasons. Firstly, as our primary interests lie in testing possible mediating relationships between various socio-demographic, clinical and familial variables in informing participants’ genetic risk perceptions (pp. 3 – 4)—we make no hypothesis around direct effects between, e.g., age group and the secondary intention outcome, as well as between ethnicity and the secondary intention outcome (Fig 2; p. 7). Secondly, we conducted mediation analysis only on pathways with significant links to the outcome measures. In the case of ethnicity, for example, given all other pathways (involving BMI or comorbidities) showed no evidence of direct effects (Fig 4; p. 10), the total effects for these pathways could not be obtained.

5. *These data will give the readers some ideas as to how informative these models are and whether there are other factors that need to be captured for the unexplained variance. In the main model, what is the meaning of the dashed line and recycling lines?*

As described in the comment above, dashed lines in the model denote paths that are not statistically significant. This is already described in p. 10 of the manuscript. Curved double-headed arrows represent variance—and, in the case of CFA analysis, covariance between two variables. This is now described in p. 8 of the manuscript and p. 12 of the appendix, respectively.

6. *It looks as though some patients might have diabetes - in the model, the factor on comorbidities included diabetes but the table did not show % of people having diabetes - please clarify.*

At the point of recruitment for the questionnaire, participants did not have T2D. This was screened twice—firstly via linked EHR data in Genes & Health and secondly, via self-reported information at the eligibility screening stage for the questionnaire (this is described in p. 4 and p. 7 of our manuscript). As described in the data analysis section of the manuscript (p. 9), the comorbidities variable in the models presented in Fig 2 and Fig 4 simply integrates participants’ self-reported history of pre-diabetes and gestational diabetes as comorbidities. The exact figures for these are reported in Table 2 (p. 9).

7. *How applicable is the European PRS to an South Asian population and would this affect their analysis?*

There has been previous work developing and optimising T2D PRSs for British Bangladeshi and British Pakistani populations in Genes & Health (please refer to Hodgson et al., 2022; as referenced in the manuscript). However, as we described in p. 5, the European ancestry PRSs selected for the current analysis were found to be the best-performing scores for the prediction of T2D as of April 2023—outperforming even the scores that have been developed within Genes & Health—due to the larger number of SNPs and sample size involved in PRS construction. Thus, we think that it was the correct decision to use the more powerful PRS in this analysis to avoid compromising validity. In this case, not using the best-performing scores available might have led to an over- or under- estimation of risk for some participants in our sample—leading to the inaccurate interpretation of results, especially in relation to the mediating relationships found between participants’ real and perceived risk for T2D.

8. *More information is needed for Table 1. They should present the data distribution of the on-line survey to help readers gain some perspectives about their profiles, family background, risk perception, contemplation for change etc. These data will also help readers understand how different variables were loaded onto the latent variable in the construction of the model.*

We thank the reviewer for pointing this out. We have now included descriptive statistics on various additional variables in Table 2 for more clarity (pp. 9 – 10).

9. *The Gene and Health Consortium recruited people of Pakistan and Bangladesh descent, but why only Bangladesh was associated with T2D PRS? The author gave some background about the recruitment of participants from various settings including mosques etc, Can the authors give some ideas about the proportion of people interested in donating DNA for the Consortium during these recruitment processes? Were there any incentives involved?*

In regards to the first point made by the reviewer in this comment, we would like to clarify that the relationships between ethnicity and T2D PRSs found in our analysis were not only restricted to only participants of Bangladeshi descent. Rather, it was the way that the ethnicity variable was coded in our final model—i.e. Bangladeshi ethnicity was coded for as the indicator variable, since it was the ethnic group with the largest *n* in our sample (approximately 54%). We have now clarified this in the manuscript (p. 9). As such, the positive relationship found in our final model simply meant that being of Bangladeshi ethnicity is associated with having higher T2D PRSs, in comparison to being of Pakistani descent. We have also clarified the wording for this finding in the results section (p. 11).

As for the second part of the reviewer’s comment, we did not replicate information about the original recruitment processes in Genes & Health in great detail, as these can all be found on the Genes & Health website (<https://www.genesandhealth.org/>), as well as their published cohort profile cited in the manuscript (please refer to Finer et al., 2020). The participant information sheet available on the Genes & Health website further describes how all Stage 1 volunteers in the cohort provide consent to donate saliva samples for DNA extraction and genetic tests (<https://www.genesandhealth.org/volunteer-information>). There are no financial incentives involved in Stage 1 procedures—but participants are made aware that their information will contribute to science and further improvements in NHS care for Bangladeshi and Pakistani communities. This, however, does not exclude the possible involvement of financial incentives should participants be recalled for Stage 2 procedures—such as in this questionnaire study, where participants were reimbursed with £15 vouchers for their participation (p. 7).

Additionally, due to the nature of recruitment methods taken in Genes & Health (e.g. approaching potential volunteers in public and community settings), it has proven difficult to obtain exact figures and/or proportions surrounding recruitment rates (i.e. it is difficult to track how many people were approached over a certain period of time in public spaces vs. how many people eventually consented). As reported in the cohort profile, however, Genes & Health has successfully recruited 38,899 volunteers between April 2015 and mid-June 2019. The latest figures are now over 60,000—and this has been updated in the manuscript (p. 4).

10. *Please give more information about the construction of the PRS - how many SNPs were used and what was the meaning of -0.13?*

The T2D PRSs used in this analysis comprises 6,437,380 SNPs. The manuscript has now been updated with this information (p. 5). Additionally, as described in p. 5, these scores have been scaled following a normal distribution with mean and median of 0 and SD of 1 in the broader Genes & Health population. The reported figure of – 0.13 in Table 2 (p. 10) thus represents the mean PRS in our questionnaire sample.

11. *The authors stated that there were missing data in participants' T2D PRSs and BMI and multiple imputations were applied alongside SEM. How would this affect the result, do we have a similar model if analyses were performed in patients with full dataset only?*

We did not perform a complete case analysis, as excluding all participants with any missing data in T2D PRSs or BMI would reduce our total sample size to *n* = 347. This loss of data would severely affect power, given the complexity of our model—and would also introduce significant healthy volunteer bias into our analysis, given these variables are missing not at random. To illustrate, most of the missing data in participants' T2D PRSs and BMI can be found in younger age groups in our sample—e.g. approximately 33% of the missingness for both variables can be found in the youngest 16 to 25 year group (the highest rates of missingness across the entire sample). These younger participants are less likely to present in primary or secondary care—thus less likely to have information such as BMI recorded in their EHRs. Complete case analysis would therefore not be appropriate nor powered enough to assess model fit—and would also likely lead to biased estimates in our SEM analysis. For these reasons, we have not presented this information in our manuscript—but would be happy to supply this for reviewers’ reference, if needed. Additionally, to demonstrate the validity of our imputations, we have now included further details surrounding our imputation diagnostics—comparing the observed and imputed values in T2D PRSs and BMI—in the appendix (S6 Appendix; pp. 15 – 16).

12. *In the supplementary text, please clarify the statement: Based on the original paper from which the primary intention outcome in our questionnaire was extracted, the authors estimated that a minimum sample size of 385 was needed to reach a power of 80%—with a confidence interval of 5%, a confidence level of 95% and SD of 0.05 [1] - did the author mean they expected only 5% of people will have a primary intention outcome of getting tested - please clarify.*

The 5% figure here represents the confidence interval—or, perhaps more precisely, the “margin of error” in questionnaire or survey research. This can be seen as the deviation allowed on questionnaire results for a particular sample—in other words, the required precision level, or a range of values above and below the actual results from a questionnaire (in principle a confidence “interval”, but for ordinal variables such as our primary outcome). To illustrate with an example, in our questionnaire, approximately 57% of the participants responded that they were “extremely likely” to seek advice to prevent or delay T2D, if a genetic test showed that they were at above-average risk for the condition. With a margin of error of 5%, we can therefore be 95% confident that between 52% to 62% of individuals in the general population would select the same answer. We have now updated the terminology in the appendix to avoid any misunderstanding (p. 1).

13. *There should be more discussion on the findings rather than just a general statement that a family and system approach will be needed, e.g. the willingness to pay and SES as suggested by the model or surveys.*

We thank the reviewer for this suggestion and agree that further interpretations are needed to emphasise the role of upstream determinants such as participants’ socio-demographic backgrounds—especially given the findings from our mediation analysis. We have already presented some of these discussions in our manuscript (pp. 12 – 13)—highlighting the need for any future efforts integrating large-scale genetic testing on a population level to account for the social determinants of health, in order to fully maximise the utility and benefits of genetic risk tools. The fact that participants from lower income households in our sample reported less interest in genetic testing largely echo the gaps in health-related perceptions and behaviours that may exist between different socio-demographic backgrounds—and should raise awareness about potential challenges to the equitable delivery and management of genetic risk tools. Whether due to a lack of awareness or understanding around the uses or purposes of genetic testing—or even perceived challenges or barriers to access health services—such differences point to the influence that characteristics linked to deprivation can have in driving health inequities (p. 12). We have now built on these interpretations by raising more specific points around the costs of genetic testing (pp. 12 – 13)—and also by emphasising the need for further funding and development in existing health systems to ensure that the costs of any such services remain accessible to the public for equitable use (p. 13).

*All in all, an interesting paper but more details and clarification are needed. The paper will also benefit from a formal statistical review.*

We hope to have addressed all concerns raised by the reviewers. In regards to the final point made about statistical review, we would like to especially thank Reviewer 1 for their valuable input, given their role as statistical reviewer.

Kind regards,

Law et al.
