# Supplementary material for: Perceived risk of type 2 diabetes: Using linked genomic, clinical and questionnaire data to understand the potential use of genetic risk tools in British South Asians
Source: PLOS Glob Public Health. 2025 Mar 31;5(3):e0004274. doi: 10.1371/journal.pgph.0004274 (PMC11957276; doi:10.1371/journal.pgph.0004274)
Supplement: Attachment — Submitted filename: response_letter.docx [file pgph.0004274.s010.docx]

Dear editor and reviewer,

Thank you for your time spent on our manuscript entitled “Perceived risk of type 2 diabetes: Using linked genomic, clinical and questionnaire data to understand the potential use of genetic risk tools in British South Asians”.

The authors would like to thank the reviewer for their valuable additional feedback—which has helped us refine our manuscript further. In particular, we are grateful for their appreciation of our study as an important addition to the literature. As described in our manuscript, the novelty of our work lies in integrating various data sources to examine factors that may have a role in informing genetic risk perceptions surrounding T2D. This work has particular importance for Bangladeshi and Pakistani populations so disproportionately affected by the condition—yet so underrepresented in genomics research.

We have addressed all comments and suggestions raised by the reviewer in our revised manuscript (please see the tracked version of our submission to view specific changes). Some of the major changes we have made include: 1) updating Table 2 with further details as suggested by the reviewer and 2) including additional figures illustrating specific mediating pathways tested in a new section in our Supporting Information. A point-by-point response to the reviewer comments can be found below:

***Reviewer comments***

*I received this manuscript to review from PLOS Global Public Health and was not a reviewer for PLOS Medicine. My expertise is genetics.*

*The authors have already had three insightful reviews from PLOS Medicine, so I will not reiterate the general material about the paper, and will focus on responses to the reviewers’ concerns, and my additional comments.*

*I enjoyed reading the paper – it makes an important addition to the literature of this field and is a good fit for PLOS Global Public Health. It provides an insightful perspective on models for risk for T2D and identifies important factors for using genetics to reduce risk.*

*The authors have responded thoroughly and appropriately to the original reviewers’ comments, improving the completeness and the quality of the paper. I have only a few minor additional comments and suggestions.*

1. *A major concern from all three reviewers was the low response rate in the questionnaire – the authors have explained this carefully and performed comparative analysis where possible. This is not a major concern for me.*

We thank the reviewer for acknowledging this—and we are pleased that the additional information and justification that we have supplied in our previous revisions have sufficiently accounted for the response rate.

2. *Table 2 is a valuable source of information about the study, participants and the data to be analysed. I suggest*
   a. *Reference groups for multilevel variables, should be indicated, for easy interpretation of the beta values reported.*

Details on the multilevel variables that are included in our SEM analysis have now been specified in Table 2 (pp. 9 and 10). Please note that this is not applicable for the diabetes history variable, as at the stage of the SEM analysis, participants’ self-reported diabetes history is combined with the number of other comorbidities extracted from their EHRs to form a continuous variable (this is described in p. 9 of our manuscript).

b. *The number of missing values are added*

Missing data for both variables T2D PRSs and BMI have now also been included in Table 2.

3. *In Table 2, I also lacked sufficient information to interpret the values on questionnaire items that have been added, e.g what does the mean of 11.9 (SE 1.66) mean for ‘Perceived control’ and how does this relate to the four questions in Part 6 of the questionnaire? Some information on coding to link between the questionnaire and the summary information would be useful. Apologies if I missed it.*

We would like to clarify that the measures of genetic risk perceptions—as well as the primary intention outcome—included in the questionnaire were all originally defined as continuous measures and scored in a numerical form (please see pp. 2 to 4 in S2 Appendix). As such, the means and standard deviations displayed for the questionnaire items in Table 2 relate to participants’ scores on these measures of genetic risk perceptions in their original, numerical form (prior to CFA analysis). We have included a brief description of this in p. 9 of our manuscript, before presenting Table 2—and have now refined this statement slightly for clarity. Additionally, we have included all details on scoring methods for all questionnaire items in S2 Appendix. To illustrate with the example provided by the reviewer, this means that the mean of 11.9 and standard *deviation* of 1.66 reported for perceived control relates to the sample mean and standard deviation on the combined scores across all four questions in the perceived control section of the questionnaire (“Overall, I feel that I am able to control my health”; “If a genetic test tells me that I have an above-average risk for type 2 diabetes, then I would think that type 2 diabetes cannot be prevented” etc.).

4. *The mediation effects are very important but are easier to interpret in a figure than in text. Is it possible to add an additional figure illustrating these results?*

We thank the reviewer for this reflection and agree with the point raised. Due to the length of the manuscript, we have included these additional figures in S7 Appendix in our Supporting information (S7 to S10 Fig, pp. 17 and 18). We have also updated our main text to reflect this addition (pp. 11 and 12 in the manuscript).

5. *My one area of concern – also highlighted in limitations – was the discrepancy between the low household income (<£18k) and the high level of education attainment (**college / university degree). The authors postulate reasonable explanations of immigration effects, or lack of translation of education levels in Discussion. A more worrying possibility is data quality, given these two response are the first item on each of those questions. Could the authors reassure us that there was no evidence of ‘lazy’ responding to the questionnaire, with (e.g.) the first item selected in all questions. I assume this type of QA is possible, and has been performed.*

We share the reviewer’s concerns surrounding possible response bias. On one hand, we unfortunately do not have the necessary timestamp data on participants’ responses to check if these high rates of specific categories reported for education and income levels were due to participants looking to answer all questions as quickly as possible (we have timestamp data on when the participants began answering the questionnaire from REDCap—but no timestamp data for when they completed the questionnaire). However, we have made some preliminary comparisons of various genetic risk perception measures between these demographic categories—which reveal no significant trends, in terms of first option selection. Taking our measure on perceived risk as an example, there was no indication that there were high rates of participants who reported coming from low income households and/or being college or university educated who also selected the first option on the perceived risk measure (“Almost no risk”). In the overall sample, 6.55% of the participants selected this first option. In the subsample reporting being college or university educated, 4.41% chose this option. In those reporting an annual household income below £18,000, 5.95% chose this option. We hope this is able to provide some reassurance—but appreciate that timestamp data would have provided a more systematic check.

We hope to have addressed all questions and concerns raised by the reviewer in our revised version of the manuscript. Thank you for your time and consideration.

Kind regards,

Law et al.
