# Supplementary material for: Perceived risk of type 2 diabetes: Using linked genomic, clinical and questionnaire data to understand the potential use of genetic risk tools in British South Asians
Source: PLOS Glob Public Health. 2025 Mar 31;5(3):e0004274. doi: 10.1371/journal.pgph.0004274 (PMC11957276; doi:10.1371/journal.pgph.0004274)
Supplement: Attachment — Submitted filename: response_letter_auresp_2.docx [file pgph.0004274.s011.docx]

Dear editor and reviewers,

Thank you for your time spent on our manuscript entitled “*Perceived risk of type 2 diabetes: Using linked genomic, clinical and questionnaire data to understand the potential use of genetic risk tools in British South Asians*”.

We are pleased to see that all comments raised by Reviewer #1 has been satisfactorily addressed—and we thank Reviewer #1 again for their valuable feedback. We would also like to thank Reviewer #2 in this round of reviews for their detailed additional comments—which has helped us refine our manuscript further. We are grateful for their appreciation of our study as an important addition to the literature. As described in our manuscript, the novelty of our work lies in integrating various data sources to examine factors that may inform genetic risk perceptions surrounding T2D. This work has particular importance for Bangladeshi and Pakistani populations so disproportionately affected by the condition—yet so underrepresented in genomics research.

We have now addressed all comments and suggestions raised by the reviewer in our revised manuscript (please see the tracked version of our submission to view specific changes). Some of the major changes we have made include: 1) providing further justification for our use of multiple imputation (specifically predictive mean matching) in our SEM analysis; and 2) updating wording across various tables and figures as suggested by the reviewer to reflect consistency in variable names. A point-by-point response to the reviewer comments can be found below:

***Reviewer comments***

1. *Abstract and introduction: The authors summarize the main research question and key findings and clearly outline the gap in the literature that the study addresses: assessing study participants’ intention to implement behavioral health changes when they are provided information about their genetic predisposition to developing T2D.*

- *Intro, paragraph 1, last sentence states “these findings demonstrate the unique benefit of tools such as PRSs to aid in early disease identification and management.” However, a more accurate characterization would be “to aid in identifying the degree of predisposition that individuals may have conferred by their genotypes at established T2D-associated genetic loci.”*

We thank the reviewer for this suggestion. This sentence has now been updated to reflect more accurate wording on p. 3.

- *Intro, paragraph 2: the points made in the sentence citing reference #18 are well taken; however, the sentence is exceedingly long and it is not clear the crux of the sentence. Recommend breaking into two separate statements and going into further detail about the research cited in reference #18.*

The long sentence has now been separated and explained in greater detail (pp. 3 to 4). This is, however, in paragraph 3 of the introduction section instead of paragraph 2, where reference #18 is—we believe there is a typo in the reviewer’s comment above.

- *Intro, paragraph 4, last sentence: similar to the aforementioned sentence, the sentence as written is too long. Would suggest breaking into two sentences and structuring the main objectives as “specific aims.”*

This section on p. 4 has now been rephrased for brevity and clarity.

- *The introduction does not discuss the* *relative utility of PRSs versus that of “clinical and/or lifestyle-related risk factors that conventional clinical risk models rely on” for predicting the prevalence and incidence of T2D*

The authors thank the reviewer for this reflection. Specific results about the relative utility of PRSs compared to QDiabetes in British Bangladeshi and British Pakistani populations can be found in the Genes & Health T2D PRS paper referenced (Reference #2). For example, when compared to a QDiabetes model providing risk estimations based on individuals’ age, ethnicity, family history, comorbidities and prescribed medications—the authors’ integrated risk tool (IRT) combining PRSs was able to generate a net reclassification improvement (NRI) of 3.22%. It was also noted in the paper that positive NRIs observed with the IRT were primarily driven by the improved reclassification of younger individuals from low to high risk in the Genes & Health cohort—NRI 5.6% in individuals under the age of 40, deemed low risk by QDiabetes alone.

Whilst we agree that it is important to emphasise these potential benefits that PRSs can offer when compared to clinical models such as QDiabetes, we feel it sufficient to discuss this in broader terms—which we have done in paragraph 1 of the introduction (p. 3). The level of detail surrounding NRIs, for example, may not fall within the scope of this paper—given our focus is more on the clinical utility of PRSs from a behavioural science perspective. However, if the reviewer still deems this as important information to include, we will be happy to integrate the above description in a revision—and will report this in the first paragraph of our introduction.

2. *Results: The figures and table captions are complete and accurate, with appropriate presentation for the types of data included. The figures and tables also support the authors’ stated findings.*
   - *Table 1: Would clarify the meaning “perceived control” and “family health behaviours” in the table itself or the figure legend. Would keep language regarding* *“primary intention” and “secondary intention” vs. “secondary interest” consistent across the manuscript and its tables/figures. Would use phrase “history of T2D” rather than “T2D history.” Also, did analyses account for history of gestational diabetes in family members?*

We have now included some further explanation for these questionnaire measures in the main text above Table 1 (p. 5)—which we hope will be sufficient. For purposes of consistency and brevity, we think it best to retain the wording in Table 1 to reflect the variable names that are used in our analysis—hence do not feel it appropriate to include in-depth explanations within the table itself. We have also signposted in the main text that details around specific items and scoring methods for these measures can be found in S2 Appendix.

Language around the “primary intention outcome” and the “secondary intention outcome” has been updated in various parts of the manuscript. Wording around “T2D history” has also been changed. Given the focus on risk of T2D, the tailored questionnaire did not account for history of gestational diabetes in participants’ family members and/or close social contacts.

- *Table 2: Would recommend indenting the individual variables within each category of variable, or coloring the row for each category title differently for easier* *legibility.*

We thank the reviewer for this suggestion—indents have now been included in Table 2 to improve readability.

- *Figure 2, Figure 3, Figure 4: Why was “Bangladeshi ethnicity” as opposed to Pakistani ethnicity included in the hypothesized model for SEM analysis? (Figure 2). “first degree family with T2D history” should be changed to “first-degree relative with history of T2D” (Fig. 2 and 4). “Income” should be changed to “income level” to reflect the various categories of income the researchers constructed (Fig. 2 and 4). “including diabetes history” should be changed to to “history of diabetes” (Fig. 2 and 4). “Knowledge” should be clarified, either in the figure itself or in the figure legend (Fig. 2, 3, 4)*.

Across all models specified in Figures 2 to 4 (as well as in the supplementary material), being of Bangladeshi ethnicity was coded for as the indicator variable for ethnicity, as this represented the category with the larger *n* in the sample. We have already included an explanation for this in the data analysis subsection of the manuscript (p. 9).

In regards to terminology, all figures concerning our SEM analysis have now been updated with the reviewer’s suggested wording for the models presented (Figures 2 to 4 in the manuscript). Figures presented in our supplementary material have also been revised for consistency.

3. *Methods*

- *Methods section, data analysis subsection:* *the hypothesised model for SEM was insufficiently described. The inter-relationships between the various variables in the hypothesised model were insufficiently justified, and the definitions of the variables (e.g., perceived control and intention) were not clearly stated in the methods section.*

We have now included some further description of the variables measured in our questionnaire under the “Questionnaire data” subheading in our methods section (main text above Table 1, p. 5). Explanations around our hypothesised model for the SEM (as well as the inter-relationships between the various variables) have been included in the subsection “Structural equation modelling” (pp. 9 to 10). Whilst we appreciate that these details would be helpful to present earlier on in the data analysis section, much of it needs to be interpreted alongside the results from our confirmatory factor analysis (CFA)—and as such, we feel it is only appropriate to provide this explanation after describing our CFA. For now, we have included a further statement alongside Figure 2 on p. 8 (depicting our hypothesised model)—signposting readers to further details around the specified relationships between these variables that will be provided in later subsections.

- *A substantial amount of data were missing for T2D PRSs (19%) and BMI (35%). Further data justifying the use of* *multiple imputation in general, and specifically predictive mean matching, as well as possible limitations of this method, should be included in the text body.*

We have now included further justification for our use of multiple imputation in general, as well as predictive mean matching in the methods section of our manuscript (pp. 9 to 10). Possible limitations relating to the integration of multiple imputation in our SEM analysis are also mentioned in both the methods (p. 10) and discussion (p. 15) sections.

- *“T2D PRSs” and “first degree family with T2D history” were both included as variables in the SEM analysis (Fig. 2 and 4). However, potential confounding due to genetic risk that may have been conferred by, or shared with, such family members, was not discussed in this section*.

We have now included a statement on potential confounding in the methods (p. 10) and limitations (p. 15) sections of our manuscript. We have also provided justification for including both variables in our mediation analysis, building on emerging evidence on the independent and complementary effects of genetic and familial risk on inherited disease susceptibility.

4. *Discussion: The authors’ discussion raises important points, citing a range of reputable articles to support their arguments. It also somewhat overreaches in the following respects:*

- *As discussed in section 3 above, the authors do not mention the potential confounding between study participants’ T2D PRSs and family history of T2D. The discussion also does not take into account that current health systems have limited, if any, implementation of PRSs at present (the NHS’s widespread efforts to genotype patients are exceptional compared with health systems worldwide and are not necessarily representative of other health systems). There is also insufficient discussion of the current literature comparing the relative contributions of genetic and clinical variables to predicting the incidence and prevalence of T2D.*

We have now included a statement on potential confounding in the methods (p. 10) and limitations (p. 15) sections of our manuscript. We have also provided justification for including both variables in our mediation analysis, building on emerging evidence on the independent and complementary effects of genetic and familial risk on inherited disease susceptibility.

We have now also included further acknowledgment of the wider context of health systems and the challenges of integrating PRSs on a larger scale in our discussion section (p. 14). This includes information on pilot trials that have already been implemented to explore the integration of PRSs for cardiovascular disease into NHS Health Checks—the national programme offering free health checks every 5 years to adults between the ages of 40 to 74 in primary care in England. Terminology has also been updated to reflect the “future” and “potential” use of PRSs, acknowledging the limited application of these tools in routine care at present (pp. 14 to 15).

As mentioned earlier, specific results about the relative utility of PRSs compared to clinical risk assessment tools such as QDiabetes in British Bangladeshi and British Pakistani populations can be found in the Genes & Health T2D PRS paper referenced in our manuscript (Reference #2). For example, when compared to a QDiabetes model providing risk estimations based on individuals’ age, ethnicity, family history, comorbidities and prescribed medications—the authors’ integrated risk tool (IRT) combining PRSs was able to generate a net reclassification improvement (NRI) of 3.22%. It was also noted in the paper that positive NRIs observed with the IRT were primarily driven by the improved reclassification of younger individuals from low to high risk in the Genes & Health cohort—NRI 5.6% in individuals under the age of 40, deemed low risk by QDiabetes alone. Whilst we agree that it is important to emphasise these potential benefits that PRSs can offer when compared to clinical models such as QDiabetes, we feel it sufficient to discuss this in broader terms—which we have done in paragraph 1 of the introduction (p. 3). The level of detail surrounding NRIs, for example, may not fall within the scope of this paper—given our focus is more on the clinical utility of PRSs from a behavioural science perspective. However, if the reviewer still deems this as important information to include, we will be happy to integrate the above description in a revision—and will report this in the first paragraph of our introduction.

We also wish to point out that the specificity of our work with a South Asian cohort adds an additional layer of complexity—as most work conducted on integrating PRSs with clinical risk assessment tools thus far has been largely focused on White European populations. Ongoing work to improve diversity and representation in genomics research can help address these challenges—but it means generalisable data on the application of PRSs for diverse populations is, at present, limited. While our work serves to highlight these issues around equitable use, the future large-scale integration of these tools with clinical risk assessment tools will necessitate continuous updates to existing risk prediction models, ensuring that the additional genetic data is accurately embedded and taking into account any updated epidemiological information in diverse populations. These are all valid concerns around the implementation of PRSs—but we again feel this to be slightly beyond the scope of this paper, where the focus is more on clinical utility from a behavioural science perspective.

- *Limitations: The authors address representativeness of the study population as well as categorization of income levels as issues affecting generalizability and external validity of the study. However, the current manuscript does not discuss limitations of the methods used in the study (see under section 3 above). The discussion section also does not cite publications that demonstrate that “PRSs often demonstrate the strongest clinical utility in younger populations.”*

Possible limitations relating to the integration of multiple imputation in our SEM analysis are now presented in both the methods (p. 10) and the discussion (p. 15) sections of our manuscript. Relevant references around the utility of PRSs in younger populations have now also been added in the statement referenced by the reviewer (p. 15).

- *Discussion paragraph 4: Sentence 2, regarding the mediation of the relationship between participants’ “perceived and actual risk of T2D” overreaches. T2D PRSs do not necessarily represent “actual risk of T2D”; would use the term “T2D PRSs” instead, and throughout the manuscript, instead. Would also posit instead of stating that “this highlights the impact that previous experiences with T2D in the family context can have on how individuals think about their own risk.” Sentence 3 is very long - would split into two*.

These suggestions have now all been integrated into our manuscript. Wording around T2D PRSs representing “actual risk” have been removed or rephrased throughout the paper—and longer sentences pointed out by the reviewer have been separated.

We hope to have addressed all questions and concerns raised by the reviewer in our revised version of the manuscript. Thank you again for your time and consideration.

Kind regards,

Law et al.
